# What Can Neuromarketing Tell Us about Food Packaging?

**DOI:** 10.3390/foods9121856

**Published:** 2020-12-12

**Authors:** Ingrit Moya, Jesús García-Madariaga, María-Francisca Blasco

**Affiliations:** Department of Management and Marketing, Complutense University of Madrid; 28003 Madrid, Spain; ingritvm@ucm.es (I.M.); fblasco@ucm.es (M.-F.B.)

**Keywords:** neuromarketing, food packaging, packaging physical appearance, food consumers’ reactions, validity, reliability, electroencephalography, galvanic skin response, eye-tracking

## Abstract

Packaging is a powerful tool for brands, which can not only catch consumers’ attention but also influence their purchase decisions. The application of neuromarketing techniques to the study of food packaging has recently gained considerable popularity both in academia and practice, but there are still some concerns about the methods and metrics commercially offered and the interpretation of their findings. This represents the motivation of this investigation, whose objective is twofold: (1) to analyze the methodologies and measurements commonly used in neuromarketing commercial research on packaging, and (2) to examine the extent to which the results of food packaging studies applying neuromarketing techniques can be reproduced under similar methodologies. Obtained results shed light on the application of neuromarketing techniques in the evaluation of food packaging and reveal that neuromarketing and declarative methodologies are complementary, and its combination may strengthen the studies’ results. Additionally, this study highlights the importance of having a framework that improves the validity and reliability of neuromarketing studies to eradicate mistrust toward the discipline and provide brands with valuable insights into food packing design.

## 1. Introduction

While touring supermarket shelves, each consumer may pass up to 300 different products per minute [1]. This means that brands’ opportunities to catch consumers’ attention are very limited. Competing in such an overstimulated context entails a huge challenge for brands, and to face it, packaging appears to be a good weapon.

The importance of packaging in marketing strategies has been extensively studied since packaging not only is the container to hold, protect, preserve and facilitate the handling and commercialization of products [2], but it also communicates brand identity, attracts consumers’ attention and helps to position the product within a concrete category [3]. Moreover, packaging is especially important in generating added value for products [4] and influencing consumers’ shopping behavior [5,6,7].

Some previous research on packaging has emphasized the multifunctionality of packaging [1,8,9,10,11], whereas other works have studied how the different elements of packaging (color, shape, size, images, etc.) can influence consumer behavior [7,12,13,14,15]. The conclusion of most studies confirmed that packaging is a critical factor in the consumer decision-making process because it can influence consumers when they are deciding what to purchase [16]. Therefore, beyond being a powerful communication vehicle for brands [17], packaging is a strategic tool that favors product identification and differentiation by breaking through the competitive clutter in a store or supermarket [18].

Due to the importance of the food industry at the global level, the analysis of the factors that drive purchase intent is of vital importance [19]. According to studies on the influence of food product packaging in consumers’ purchase decisions, the selection of food in supermarkets is a complex process determined by sensory and non-sensory attributes [20], and it is affected by diverse factors, such as the involvement level and time pressures [7]. Both packaging attributes and purchase context characteristics act by influencing consumers’ perceptions of the products, which conditions their evaluation of them and, consequently, affects the purchase decision.

Given the above, consumers’ perception packaging is essential, especially regarding food products where people usually have to choose among relatively similar products [3]. By understanding how consumers perceive, evaluate and choose food products, the industry will optimize its packaging design and achieve an added value that can contribute to brands’ business strategies [9].

To improve our understanding, a rapidly growing area of research interest is neuromarketing, whose main advantage lies in the possibility of using neuroscience techniques to evaluate the product packaging more directly, that is, without having to rely on what consumers say about which packaging they prefer [21]. By using techniques such as functional magnetic resonance imaging (fMRI), electroencephalography (EEG), galvanic skin response (GSR) and/or eye-tracking (ET), neuromarketing measures processes and behaviors that are outside of the individuals’ awareness [22].

The application of neuromarketing techniques to packaging has recently gained considerable popularity both in academia and practice. Evidence of this can be seen in companies such as Nielsen, Kantar or Ipsos, which have included neuromarketing in their commercial offers. However, despite the development of the discipline, there is a concern with some commercial neuromarketing studies that have been conducted to date regarding the methods and metrics offered and the interpretation of their findings [23]. According to Spence [24], some neuromarketing companies often go beyond the conclusions that can legitimately be drawn from the data. Fisher et al. [25] noted that some neuromarketing companies make questionable claims without evidence-based citations, and Hensel et al. [26] highlighted that claims and findings extrapolated from neuromarketing studies are not always grounded on valid scientific methodologies. 

Thus, whereas academic studies are based on rigorous protocols and are subject to a scientific approach that involves endorsement of ethics committees and are published after a peer review system [27], the validity of methods and metrics in commercial studies has been a critical issue since the earliest days of the field [23,28]. 

Regarding the evaluation of food packaging, Spence et al. [24] mentioned that, in addition to the uncertainty surrounding the methods of some commercial studies in neuromarketing, there are difficulties in providing brands with relevant answers, especially in studies that go beyond the evaluation of the visual aspects of packaging. Therefore, it is not only it is important to analyze and disseminate the methodologies and measurements used in commercial research on packaging, but also to provide evidence of their validity and reliability [23,29,30].

Taking into account the importance of identifying whether neuromarketing methods are valuable and whether these measures actually translate into real-life success, the objective of the present study is twofold: (1) to analyze the methodologies and measurements commonly used in neuromarketing commercial research on packaging, and (2) to contrast whether neuromarketing studies are consistent concerning the results obtained, and therefore to examine the extent to which the results of food packaging studies, applying neuromarketing techniques, can be reproduced under similar methodologies.

Since neuromarketing refers to “practitioner and commercial interest in neurophysiological tools to conduct company-specific market research” [31] (p. 651), commercial studies on food packaging are usually oriented to comparing two or more packaging or analyze how different versions of the same packaging work [23]. The main goal of commercial neuromarketing studies is to measure crucial aspects for understanding consumer behavior, not only in the unconscious domain (attention, emotional response, and memory) but also regarding the declarative one (attitudes and preferences) [30,31] in order to detect differences that allow brands to identify the best strategies in the packaging domain.

Hence, the hypotheses that guide this work establish the following:

**Hypothesis 1** (**H1**)**.**  There are differences in the participants’ attitudes, preferences, purchase intention, attention, emotional response and memory regarding the tested packaging.

**Hypothesis 2** (**H2**)**.**  The use of the same methods and metrics in two separate studies, with comparable samples, leads to the same results and conclusions.

To test the established hypotheses, we conducted two separate experiments: Study 1 was used to analyze the conscious and unconscious reactions of consumers to five different food packaging, by using a combination of neuromarketing and declarative techniques and following the methodology of a standard commercial study of neuromarketing. Study 2 was designed to examine whether neuromarketing studies were consistent with respect to the obtained results, and therefore to examine to what extent the results of the food packaging studies, applying neuromarketing techniques, could be reproduced under similar methodologies.

## 2. STUDY 1. Analysis of Conscious and Unconscious Reactions of Consumers to Food Packaging

### 2.1. Materials and Methods

Participants included 43 healthy right-handed adults (22 women-21 men) aged between 18–25 years (*M/SD* = 23.3/2.81) who were recruited to participate in the study by using convenience sampling. None of them informed us of any history of neurological or psychiatric illness, nor visual problems. In accordance with the local legislation and institutional requirements, ethical review and approval were not required for this study. However, the research was conducted in compliance with the guidance of the Helsinki Declaration. All participant signed informed consent forms before participation and received monetary compensation at the end of the experiment as a token of appreciation.

**Figure 1:**
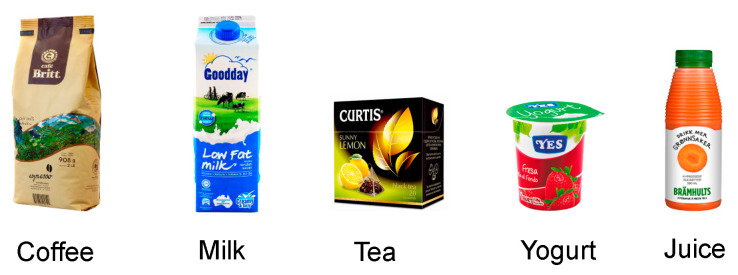
Stimuli used in Study 1.

The within-subjects experiment was based on the passive visualization of five different food packaging (coffee, tea, milk, yoghurt and juice) (See Figure 1). Product categories were selected among packages of everyday commodities and based on the following: (1) the analysis of products most often tested on previous studies [3], and (2) the results of a focus group discussion conducted with a sample of eight people aged 18–35 years. All packaging images were presented in full-color and had the same size and format. None of the brands and/or products tested in the present study used is marketed in Spain to avoid the familiarization bias.

#### 2.1.1. Data Collection

The data collection process entailed the application of two research methodologies: neuromarketing and declarative techniques. The employed neuromarketing techniques were: (1) electroencephalogram (EEG) (2) galvanic skin response (GSR) and (3) eye-tracking (ET). For the declarative techniques, a questionnaire was applied. All instruments and the measurements provided by each one are described in the following sections and can be found in Table 1.

##### Neuromarketing Techniques

- Electroencephalography (EEG)

EEG is a non-invasive technique that measures the activity of brain areas, revealing the subjects’ state of cortical activation [31]. EEG records the electrical activity of the brain expressed in five brain waves, each characterized by different frequencies and amplitudes: delta (0–4 Hz), theta (3–7 Hz), alpha (8–12 Hz), beta (13–30 Hz), and gamma (30–40 Hz), which reflect different cognitive and affective states [32]. This technique is frequently used to measure subjects’ unconscious responses to diverse marketing stimuli [33].

Although in the field of food packaging most of the studies using neuroimaging have been oriented toward identifying the neural correlates of packaging processing by using fMRI [27], EEG has been also used, especially to investigate consumers’ cognitive or affective reactions to the different dimensions of a product, especially the packaging design [15,32,34,35].

To achieve the objectives of this study, we monitored the brain signal of participants using a BitBrain Versatile EEG (12 channels, sampling rate of 256 Hz, impedances <5 Ωk).

- Galvanic Skin Response (GSR)

The GSR device measures subjects’ electrodermal activity (EDA), which is a psychophysiological indicator of their emotional arousal [36]. EDA occurs when, due to exposure to a relevant stimulus, the sweat glands increase their activity, and consequently, the skin becomes a better electrical conductor [37]. Thus, measuring EDA consists of measuring the electrical conductance, resistance, impedance, or admittance of the skin, expressed in microsiemens (μS) [38,39]. 

In the marketing context, EDA has been used in consumer research to obtain real-time data on consumers’ emotional state, captured without any verbalization [40], and specifically in the area of food research, EDA has been used to analyze the emotional impact of products and their components, among them the packaging design [41,42,43].

The GSR device used in the present study was the BitBrain GSR ring, a wireless device for real-time monitoring of EDA and cardiac activity.

- Eye-Tracking (ET)

ET technology measures eye movements composed by fixations and saccades to determine precisely where the subjects’ attention is directed [44]. Fixations correspond to periods during which the eyes remain still on an object for approximately 200–300 ms, which allows an individual to identify all details of the object. Saccades instead, are the eye movements between two fixations, lasting from approximately 40 to 50 ms [45].

The ET technique is based on the hypothesis that what people are looking at reflects the cognitive processes taking place in their mind, and, consequently, what people are looking at reflects where their attention is oriented. Thus, ET metrics are used to assess where, when, and what people look at. To obtain such information, ET operates by using an optical camera that reflects a near-infrared light onto the cornea, allowing it to identify the position of the eyes. [30].

ET is increasingly being applied in the fields of consumer research and marketing as a means of exploring how consumers process visual information [46], and it has been used in the field of packaging design evaluation for many years, essentially to establish how people explore packaging and also to identify which packaging elements are able to catch the consumers’ attention [6,15,45,46,47,48,49]. In this study, the subjects’ eye movements were recorded with a Tobii X2-30 Eye-Tracker Compact Edition (60 Hz).

#### 2.1.2. Measurements

##### Neurophysiological Metrics

- Frontal Alpha Asymmetry (FAA)

The EEG index that is most often used in neuromarketing is the FAA [31]. The underlying theory of this index suggest that the left part of the frontal cortex is involved in experiencing positive emotions, which leads to a tendency to approach stimuli perceived as desirable, while the corresponding area on the right side of the frontal cortex is involved in the processing of negative emotions and consequently related to defensive withdrawal from stimuli [50]. Therefore, FAA is assessed by comparing activation levels between comparable areas on the left and right sides of the frontal cortex [51]. More specifically, the FAA index is obtained by analyzing the alpha wave (8–12 Hz) on right (F4 and F8) and left (F3 and F7) sides of the frontal cortex and is the result of computing the power differences between the two sides (F4 and F8–F3 and F7).

Taking into account that decreases in the alpha power (alpha desynchronization) in a particular brain region are related to a higher cortical activation [52], a positive value of the FAA index will indicate a greater activation of left than of the right hemisphere [53].

As aforementioned, FAA is broadly employed in neuromarketing research since it is widely accepted as an index of approach–withdrawal attitude toward stimuli [51,54,55]. Regarding food packaging, FAA has been continuously applied to study the effects of food appearance (shape, size, color and packaging), taste and flavor on consumer emotions [56].

- Cognitive Load

The concept of cognitive load relates to the amount of mental or physical resources that people need to complete a particular task [57]. Increases in working memory due to tasks such as problem-solving or analytical reasoning are related to the EEG cognitive load index [58], which reflects the mental effort required to perform the task [59]. When the effort increases and, consequently, the demand for resources also increases, the theta band power in the frontal channels increases (synchronization), while the alpha band power in the parietal channels decreases (desynchronization). This represents the situation of visual attention and semantic tasks, which typically generate a decrease in the alpha rhythm in the prefrontal cortex [59,60,61,62].

In this research, the cognitive load was calculated according to the existing literature [59,60,61,62,63], by calculating the ratio between the theta band power in the frontal channels (F3 and F4) and the alpha band power in the parietal channels (P3 and P4).

Regarding food evaluation, some studies have examined the impact of cognitive load on the processing of packaging information [64] or even the processing of food odors [65,66]. However, to our knowledge, there are still few studies that use EEG to measure the cognitive load of consumers.

- Memory Encoding

How information is stored in memory is a question widely studied, since human experience in the world depends on memories. Memory has been studied from different points of view: cognitive, anatomical and neurophysiological [67,68,69]. For the present study, it is especially important to talk about the neurophysiological level. 

As memory is made up of a number of interrelated systems, organized structures and behavioral and cognitive correlates [70], to study it, neuroscientists have classified this unitary system into separate sections attending to its intrinsic characteristics [71]. The main classification is related to the duration of memory and divides it into short-term memory (also known as working memory) and long-term memory. 

Long-term memory is composed of declarative and non-declarative systems. Declarative memory is considered as consciously represented and retrievable and made up of memory for facts (semantic memory) and memory for events (episodic memory), whereas non-declarative memories are classified into procedural, priming, associative, as well as non-associative conditioning, and are largely unconscious [72].

Food products can be represented in semantic memory as well as in episodic memory [73]. Semantic memory stores, processes, and retrieves conceptual abstract product knowledge relevant for rational decision making, whereas episodic memory stores, processes, and retrieves information that is self-relevant and subjectively framed [72].

According to previous studies, the encoding process is reflected in the theta band (3–8 Hz) [67,68,69]. In fact, theta activity exhibits both increases and decreases during successful memory formation [60,74,75,76,77]. Osipova et al. [76] found that increased frontal theta power was observed for later-remembered versus later-forgotten stimuli, and Mitchell et al. [78] reported the presence of theta activity at frontal electrode sites during the performance of episodic memory tasks.

In the present study, global field power (GFP) was used to quantify the global activity in prefrontal channels (Fp1, Fp2, F7, F3, F4, and F8) filtered on the theta band.

- Emotional Arousal

Emotional experiences can be described by two factors: (1) valence, related to how negative or positive the experience is, and (2) arousal, which describes how calming or exciting it is [79]. Variations in arousal are associated with changes in the autonomic nervous system activity in both the sympathetic and the parasympathetic systems [34], which are expressed on physiological reactions such as increases in heart and/or respiratory rate, pupil dilatation or increases in the activity of the sweat glands [37].

As aforementioned, the GSR device measures the electrical properties of the skin, referred to as the subjects’ electrodermal activity (EDA), which is a psychophysiological indicator of their emotional arousal [36]. Because phasic skin conductance responses (SCRs) are a reliable indicator of arousal states [80], we calculated the participants’ arousal levels by calculating the amplitudes of the skin conductance response (SCR).

- Visual Attention

Attention is defined as the ability to focus on certain aspects of the environment while ignoring others [30]. ET provide direct measures of attention since it tracks subjects’ gaze when viewing diverse stimuli, providing information about how participants explore them (locations, order and duration) [81].

Eye movements are closely coupled with visual attention, making them eminent indicators of the visual attention process [45]. As a consequence, several parameters of oculomotor behavior are nowadays used in the study of packaging. Visual fixations are perhaps the most commonly used parameter when it comes to assessing where a consumer’s attention might be focused [46]. Fixations are defined as gaze patterns in which the eyes are relatively immobile, and during which the visual system is assumed to be gathering information [82]. In this sense, their frequency (number of fixations) and duration (time spent at any specific area of the stimuli) can be measured. Therefore, we used the total time that participants spent looking at each packaging (time in the area of interest (AOI)) and the number of fixations on them as indicators of the visual attention of the subjects.

##### Declarative Metrics

To evaluate participants’ attitude towards the tested packaging, a questionnaire was applied. The dependent variables were (1) appreciation, (2) perceived complexity and (3) purchase intention. 

Appreciation was assessed by using two semantic differential items: not appealing/very appealing and dislike/like. The response options were on a seven-point Likert scale. Cronbach’s alpha for this construct was 0.905. The degree of perceived complexity was operationalized on the basis of the semantic differentials: straightforward–unclear and easy to understand–difficult to understand, on a seven-point Likert scale. Cronbach’s alpha for this construct was 0.955.

Finally, two questions were used to assess the purchase intention. Responses were also given on a seven-point Likert scale, with answer options ranging from 1 (strongly disagree) to 7 (strongly agree). The internal consistency (Cronbach’s alpha) of the questions “I would consider buying this product” and “I would recommend this product” was 0.964.

### 2.2. Procedure

The experiment was developed at the Neuromarketing Laboratory of Commerce and Tourism School at the Complutense University of Madrid. The average duration for each participant was 45 min, including neurophysiological recording and the declarative questionnaire. 

After briefing participants about the protocol, the EEG and GSR devices were affixed. The ET used was fixed on the computer screen in front of participants, and to calibrate it, subjects had to follow the points on the screen with their eyes, keeping their heads static. When the ET was calibrated, the signal of EEG and GSR were checked, and if corrected, the experiment began. 

Using the SensLab software, developed by BitBrain, subjects visualized five food packaging, while their neurophysiological reactions on the three neuromarketing devices used were recorded simultaneously. The stimuli were presented individually and randomly, and the exposure time was uniform for all participants (5.000 ms).

In the second stage, once neuromarketing devices were removed, participants answered the declarative questionnaire where they had to evaluate the stimuli individually.

### 2.3. Data Analysis

A band-pass filter between 1 and 25 Hz with a four-order Butterworth filter was used to filter the raw EEG data. Then, a three-step filter pipeline was implemented: (1) to remove large amplitude artefacts, we used the artefact subspace reconstruction (ASR) [83]; (2) to separate the EEG data into independent components, we applied an independent component analysis (ICA) [84]; finally, (3) to automatically classify ICA components as artefacts, we applied the Multiple Artifact Rejection Algorithm, well known as MARA [85]. 

With the signal clean, the EEG indexes (FAA, cognitive load and memory) were computed as described in the available literature. To obtain the frequency bands, the Welch method was applied to obtain the power spectral density. Theta and alpha bands were individualized using IAF (individualized alpha frequency) analysis [86]. 

In order to obtain the EDA data, the first step was the deconvolution of recorded data to subsequently conduct the computation of tonic and phasic activity. We then applied a low-pass filter to remove the muscle noise and detect the sweating peaks in the GSR signal. Since according to the previous literature, SCR is a reliable indicator of arousal states [80], in the present study, we computed the subjects’ arousal based on the estimation of the SCR amplitudes. ET information was provided by SensLab Software and combined with the post-processed EEG and GSR signals, and the declarative data were analyzed using SPSS.

### 2.4. Results

Firstly, we analyzed the results by comparing the implicit and explicit measurements of the tested packaging. Subsequently, we carried out the analysis to identify the substrate of the identified differences. 

Regarding the neurophysiological measurements, the results of a repeated-measures ANOVA showed that the EEG memorization index yielded statistically significant differences among the different products (F (4, 164) = 2.453, *p* = 0.04). A post hoc test using the Bonferroni correction revealed differences in encoding between yoghurt (*M* = 2.27) and juice (*M* = 0.35). No statistical differences were found for the remaining EEG measures, nor for arousal.

We also found differences in visual attention measured by using ET. A repeated-measures ANOVA concluded that the different food packaging yield statistically significant differences in time in AOI (F (4, 168) = 2.370, *p* = 0.05). Post hoc tests using the Bonferroni correction revealed significant differences in time in AOI between coffee (*M* = 5.31) and juice (*M* = 4.61) packaging. Moreover, statistically significant differences were found in the number of fixations (F (4, 168) = 6.020, *p* = 0.00). A post hoc test revealed differences between juice (*M* = 5.07) and all other packaging (*M–coffee* = 7.00; *M–milk* = 6.82; *M–tea* = 7.60; *M–yoghurt* = 7.07).

Regarding the declarative measurements, there was only statistically significant differences in the perceived complexity among the tested packaging (F (3.130, 131.473) = 3.224, *p* = 0.02). According to the performed post hoc test, there were differences between milk packaging (M = 6.44) and all other packaging (*M–coffee* = 5.65; *M–tea* = 5.86; *M–yoghurt* = 6.12 *M—juice* = 5.83). It is important to note that participants scored the most complex images with one and the least complex with seven. Thus, milk packaging was categorized as easy for participants. 

To analyze the relationship between the variables, a correlation analysis was performed. Results showed three strong and positive correlations, which were statistically significant: (1) between memorization and cognitive load (*r*(42) = 0.326, *p* = 0.035), (2) between appreciation and purchase intention (*r*(43) = 0.689, *p* = 0.000) and (3) between time in AOI and the number of fixation in AOI (*r*(43) = 0.558, *p* = 0.000).

## 3. Study 2. Analysis of Reliability of Neuromarketing Food Packaging Studies

This second study was created to determine whether the neuromarketing studies were consistent with respect to the results obtained, and therefore to examine to what extent the results of food the packaging studies, applying neuromarketing techniques, could be reproduced under similar methodologies. To this end, participants were exposed to nine food products commonly consumed by the age range, belonging to three different categories. The selection of categories and products was the result of fourteen in-depth interviews and a focus group performed before the study.

### 3.1. Materials and Methods

**Figure 2:**
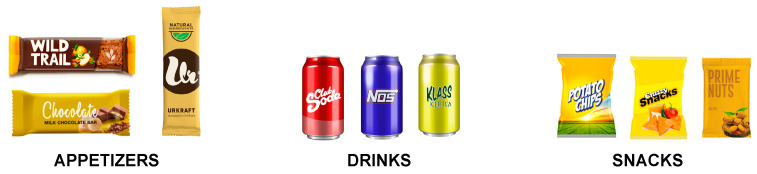
Stimuli used in Study 2.

Forty people (21 women and 19 men; age range: 18–25; *M/SD* = 22.8/2.3) were invited to participate in the study. The stimuli were nine food products belonging to three different categories: (1) soft drinks (ice tea, soda and energy drinks), (2) snacks (chips, nuts and nachos) and (3) appetizers (cookies, chocolate bars and cereal bars) (See Figure 2).

As in the first study, all participants had normal or corrected-to-normal vision, were right-handed and signed an informed consent according to the Declaration of Helsinki. In addition, at the end of the experiment, all participants received monetary compensation.

To achieve our goal of analyzing the reliability of the first study, the experimental design was the same for both studies. Thus, both instruments and measurements were the same as in the first study. The information of Section 2.1.1 Data collection, Section 2.1.2 Measurements, Section 2.2 Procedure and Section 2.3 Data analysis applies in the same way for this second study.

### 3.2. Results

Data analysis was conducted in three stages. First, we analyzed the inter-category differences to subsequently analyze the intra-category differences. Finally, we test the relationship among the dependent variables. 

Regarding the inter-category differences, a repeated-measures ANOVA determined that there were not any differences in the EEG and GSR metrics among the three tested food categories. In contrast, the same test showed statistically significant differences in visual attention, in the two ET metrics included (1) the number of fixations (F (2, 76) = 245.5, *p* = 0.000) and (2) time spent exploring the packaging (F (2, 76) = 159.3, *p* = 0.000). Post hoc tests using the Bonferroni correction revealed significant differences in both metrics between drinks and the other two categories. No statistically significant differences were found in declarative metrics.

Concerning the intra-category differences, repeated-measures ANOVA was performed to analyze the differences between the three products included in each food category. The obtained results determined statistically significant differences between products of the appetizers category in (1) the number of fixations (F (1.54, 44.62) = 34.28, *p* = 0.000) and (2) time spent exploring the packaging (F (2, 58) = 7.44, *p* = 0.001). Post hoc tests using the Bonferroni correction revealed significant differences in the number of fixations between cookies (*M* = 1.61) and cereal bars (*M* = 3.17) and chocolates (*M* = 2.95). In regard to the time spent exploring the packaging, differences were found between the three tested products (*M–cookies* = 3.00, *M–cereal bars* = 1.17, *M–chocolates* = 6.13).

The same situation was found in the category of snacks, where repeated-measures ANOVA determined statistically significant differences among the three products of category in (1) the number of fixations (F (2, 68) = 8.69, *p* = 0.000) and (2) time spent exploring the packaging (F (2, 68) = 9.28, *p* = 0.000). Post hoc tests using the Bonferroni correction revealed significant differences in the number of fixations between nachos (*M* = 4.03) and chips (*M* = 2.23) and nuts (*M* = 2.60). Regarding the time spent exploring the packaging, differences were found among nuts (*M* = 3.35), chips (*M* = 1.95) and nachos (*M* = 2.53).

There were no statistically significant differences in EEG and GSR metrics, nor in declarative metrics.

Finally, regarding the relationship among the dependent variables, two positive and statistically significant correlations were found, not only inter-category but also intra-category. Correlations between inter-categories were as follows: (1) between appreciation and purchase intention (*r*(40) = 0.680, *p* = 0.000), and (2) between time in AOI and the number of fixations in AOI (*r*(40) = 0.749, *p* = 0.000). Concerning the relationship between memorization and cognitive load, a positive and statistically significant correlation was found only between the products belonging to the snacks category (*r*(38) = 0.325, *p* = 0.047).

## 4. Discussion

Based on the obtained results, the first point to address is the lack of significant differences between packaging for most of the metrics. Since, according to our first hypothesis, we expected that there would be differences in the participants’ attitudes, preferences, purchase intention, attention, emotional response and memory among the tested packaging, we can conclude that the hypothesis could not be confirmed. Regarding this point, there are some explanations that must be addressed.

A technical explanation for the lack of statistically significant differences in the EEG metrics might be the limited nature of EEG as a measure of brain processes [87]. EEG only reveals the synchronized local field potentials of well-aligned cortical pyramidal neurons, because it is susceptible to only a subset of electrical events in the brain [88]. Moreover, some evidence suggests that activation in subcortical areas is unlikely to contribute directly to the scalp EEG signal [89]. Unfortunately, most of the higher-order psychological and emotional areas of the brain are located at a deeper level within the brain [90]. Thus, in some contexts, it is difficult to obtain signals from those deeper portions of the brain.

On the other hand, even though EEG is well known as a useful tool to analyze consumer preferences and decision making by measuring the cortical activity elicited by the brain [91], EEG recordings in an experimental environment during a certain cognitive task require stimuli that can induce significant changes in the neuronal networks that are under investigation [92]. This means that obtaining significant results from EEG depends on the extent to which the tested stimuli can provoke neural responses.

Furthermore, individual differences in brain processes can either be stable dispositions evident in some situations or a characteristic response to specific stimuli [87,93]. In this sense, the lack of stimulation may be the cause of the failure to find significant differences in the EEG signal. 

Moreover, as aforementioned, while touring supermarket shelves, each consumer may pass up to 300 different products per minute [1]. As the stimuli used in the present study were packages of everyday products that the participants are used to seeing, it was difficult for a specific package to stand out, and this may justify the lack of significant differences not only in the neurophysiological measurements, but also in declarative measurements. 

Moreover, as Mojet et al. [94] affirm, the implicit measurements deliver product information that is not always related to the consumers’ preferences. Although the visual aspects of packaging design are undoubtedly highly important, it is crucial to realize that packaging is inherently multisensory and, therefore, neglecting the influence of other sensory components of packaging in consumers’ evaluation of them may limit the scope of studies on food packaging [27,90].

On the other hand, taking into account the fact that our second hypothesis stated that the use of the same methods and metrics in two separate studies, with comparable samples, leads to the same results and conclusions, it is worth analyzing the results obtained in this matter.

Golafshani [95] defined reliability as the extent to which results of a study can be reproduced using a similar methodology. Therefore, the concept of reliability is related to whether a given metric is consistently producing the same response, and thereby, the same conclusions [23]. Regarding the validity, Ramsøy [23] highlighted that it regards whether a given claim can be supported and clarified that it is about contrasting if a specific measure works beyond a controlled lab environment.

Testing the validity and reliability of a study requires the replication of all experimental conditions in comparable samples, but this replication may fail due to the intrinsic characteristics of the instruments. In the case of neuromarketing, and specifically, regarding EEG, some metrics are sensitive to responses that are not considered its main target [96]. This happens because the same brain structure can be engaged in different processes, depending on the task performed, the context, and its connectivity with other brain areas [97]. This situation makes it difficult to identify in which cases the obtained response is associated with a specific process.

In this context, triangulation seems to be a suitable strategy for improving the reliability of research or evaluation of findings [95]. However, triangulation requires that a given metric corresponds with other metrics (internal consistency reliability). Unfortunately, most of the commercial studies in neuromarketing, and also the present study, do not use different methods to measure a single construct. However, even in basic research, further research is needed to ensure the correspondence between metrics and, consequently, their specificity. 

In this vein, and in light of the obtained results, it is evident that neuromarketing methods do have limitations, and this is why the second hypothesis stated in the present study is not confirmed. Our findings show the difficulties associated with executing neuromarketing studies and evidence that results should be interpreted with caution. The interpretation of neuromarketing results should take into consideration the complexity of the experimental design, the nature of the stimuli, the paradigm used and how other method-induced variances may impact the results [98]. It could be a mistake to progress directly to the numbers while ignoring the context of the study.

Moreover, our results reveal that many diverse factors interact in the evaluation of food packaging, and more research is needed to understand the role of each of them in the decision process. Besides, our findings support the idea that it is crucial to ensure a robust and valid science and commercialization of neuromarketing and support other authors’ claims regarding the importance of being rigorous about ensuring the validity of neuromarketing approaches and measures [23,28,99]. 

Finally, and going beyond the hypothesis that supports the present study, it is worth analyzing some other interesting findings. The first point to highlight in this context is the correlation found in the first study between cognitive load index and memory encoding, which was repeated in the snacks category. The main justification for this convergence is that both indexes are based on the synchronization of theta band in frontal brain. Frontal theta power has previously been delineated as a marker of mental effort and increased attentional demands [100]. Additionally, frontal theta activity has been related to successful memory formation [67,68,69,101]. 

However, some authors have also found a deeper relationship between these indexes, because theta oscillations from the frontal cortex are associated with the neural basis of short-term or working memory, more specifically when encoding and retaining of information [60,61,102,103,104].

Another reasonable explanation is found in the analysis of how the general sensory and cognitive pathway functions during the interpretation of a visualization. When a subject is exposed to any image, it is first processed by the visual system and then organized and evaluated by the working memory and cognition centers. Then, prior knowledge is used to determine the appropriate cognitive schema for data interpretation [62]. 

Previous studies suggest that theta plays a role in context updating [105]. The correlation found in the present study is consistent given that successful memory encoding depends on the organizing and evaluation of images, which is related to cognitive load and a correct context updating, which, in turn, are related to memorization index.

The second point to review is the results obtained for the two visual attention metrics: time spent exploring the packaging and the number of fixations on it, which are uniform for both studies carried out. Although a strong positive correlation was found between both metrics, it was not possible to correlate their behavior with the other dependent variables. This means that no pattern was found in these metrics. 

According to Ares et al. [12], an increase in the fixation duration on the packaging suggests that consumers did not comprehensively assess the information presented, and that the increase is an indication that consumers had to perform more intensive information processing, as it was more difficult to extract the information they needed due to its greater density. Furthermore, Gholami et al. [91] pointed out that the product itself may not significantly enthuse consumers. Therefore, consumer attention may increase with additional influential features (colors, images, texts, etc.) and also could be drawn to familiar marketing stimuli, because consumers prefer to focus on what they know at the expense of new information.

While other authors point out that ET is not suitable to measure how difficult information processing is for consumers [15], ET metrics might instead be related to the good feeling evoked by the stimuli. Based on the aforementioned, further research is needed to delve deeper into this issue and go beyond the differences among the metrics to identify the determinants of their behavior.

The final topic to analyze is the perceived complexity. Previous studies link the cognitive load index to cognitive performance, suggesting that increases in the oscillations in the alpha band could be an indication of a cognitive overload caused by a too-complex visualization task [60,63]. On the basis of those findings, packaging perceived as too complex could be expected to yield higher scores in the cognitive load index, and, consequently, there may be a significant correlation between the neural cognitive load index and the declared perceived complexity. However, such a relationship was not found.

According to Greenwald and Farnham [106], divergences between implicit and explicit measures might occur when self-reports are inaccurate due to response biases, whereas implicit measures are assumed to be less affected or even unaffected by such biases. Heyligen [107] noticed that the perceived visual complexity is a function of the quantity and range of objects perceived by the subject in the image. In the same line, Harper et al. [108] remarked that complexity perception depends on the subjective evaluation of the perceived parts in the scene, familiarity with the scene, and existing knowledge of objects inside the scene. 

Taking into account that self-reported perceived complexity is the result of the participants’ subjective evaluation, whereas cognitive load index is the reflection of the cognitive resources needed to process a stimulus [62], the findings of the present study are consistent with the existing literature on this topic.

## 5. Conclusions

Although the use of neuroscience insights and methods has been shown to improve our understanding of consumer behavior [99], the use of a multi-method approach is highly recommendable to strengthen the results of using neuroscience methods [98]. 

The present study provides evidence of how, through the integration of declarative and neuromarketing techniques, we gain a more holistic approach to the consumer reactions to food packaging evaluation. However, we also provide evidence regarding how difficult and confusing the analysis and interpretation of neuromarketing data might be, and support Anderson et al. [62] regarding the notion that neurophysiological data analysis requires training and expertise. 

In addition, although the fact that we did not find significant differences in some metrics may appear to be a weakness at first, this is an interesting finding itself, because it is in the same line as Lin et al. [98], who highlighted the need to take into consideration the experimental design, the nature of the stimuli and the context of subjects to obtain meaningful results. In fact, in view of the results obtained, and taking into account the subtle differences found between the tested packaging, it would be interesting to conduct another experimental paradigm instead of a passive visualization.

Undoubtedly, the application of neuroscience techniques in food packaging research represents a huge step, but in order to provide relevant answers for brands, further research is needed to understand how the different elements of packaging influence the product evaluation and consequently the purchase decision. 

In addition, despite the large number of scholars studying food packaging by using neuromarketing tools (see [31]), this is a young discipline. Consequently, its theoretical, empirical and practical field is still in development. Therefore, from the academic side, we must provide a validation framework that helps neuromarketing companies to provide metrics capable of responding to what they are supposed to measure. Only through joint efforts to achieve standardization in this discipline will we eradicate the overpromising and under-delivering that have been affecting the reputation of our area.

## 6. Limitations and Further Research

With reference to limitations, a common criticism against the use of neuroscience methods to study consumer behavior is the use of relatively small sample sizes, perceived as resulting in low statistical power [98]. However, previous research has shown that consumer neuroscience studies are capable of producing meaningful insights and predictive results using small sample sizes [109,110]. Moreover, the size of our samples is even bigger than that indicated by Desmond and Glover [111], who estimate that up to 25 subjects are able to ensure 80 per cent statistical power.

On the other hand, as stated by Mast and Zaltman [112], a great deal of experimental work is required to identify which set of problems are best addressed using neuromarketing and how the information provided by its techniques could be better complement existing methods. In this sense, the present study is a starting point for further research that will demonstrate what kind of issues related to packaging could be better addressed through the use of neuromarketing. 

Furthermore, given that this is an exploratory study using a convenience sample of people between the ages of 18 and 25, it would be interesting to conduct more research to show if different results can be found in other age groups. Additionally, it would be productive to analyze how the use of larger samples and/or other product categories can influence the results.

Finally, although our experimental approach was motivated by the desire to test the reliability of commercial studies of neuromarketing, the fact that the tested packagings were highly familiar to participants, in addition to the experimental design based on a spectral EEG, provided no significant results. Therefore, further research is needed to test whether using a stimuli-induced reaction results might provide statistically significant differences. Furthermore, it is extremely important to advance in the study of the validity and reliability of neuromarketing studies to eradicate mistrust around the discipline and provide brands with valuable insights into food packing design.

## Figures and Tables

**Table 1:** Instruments and measurements.

	Instruments	Measurements
Neuromarketing techniques	EEG ^1^	Frontal alpha asymmetry (FAA)
		Cognitive load
		Memory encoding
	GSR ^2^	Emotional arousal
	ET ^3^	Time spent in the AOI ^4^
		Number of fixations in AOI ^4^
Declarative methodologies	Questionnaire	Appreciation
		Perceived complexity
		Purchase intention

^1^ EEG: electroencephalogram. ^2^ GSR: galvanic skin response. ^3^ ET: eye-tracking. ^4^ AOI: area of interest.
